# Public perceptions of digital fashion: An analysis of sentiment and Latent Dirichlet Allocation topic modeling

**DOI:** 10.3389/fpsyg.2022.986838

**Published:** 2022-12-28

**Authors:** Yixin Zou, Ding-Bang Luh, Shizhu Lu

**Affiliations:** School of Art and Design, Guangdong University of Technology, Guangzhou, China

**Keywords:** digital fashion, NFT, sentiment analysis, (LDA) topic modeling, virtual fashion, public perceptions, digital fashion trends

## Abstract

Since digital technology has had a significant impact on the fashion industry, digital fashion has become a hot topic in today’s society. Currently, research on digital fashion is focused on the transformation of enterprise marketing strategies and the discussion of digital technology. Despite this, the current study does not include an analysis of the audience’s emotional and cognitive responses to digital fashion on social networking platforms. A comprehensive analysis and discussion of 52,891 posts about digital fashion and virtual fashion published on social networking sites was conducted using k-means clustering analysis, Latent Dirichlet Allocation (LDA) topic modeling, and sentiment analysis in this study. The study examines the public’s perception and hot topics about digital fashion, as well as the industry’s development situation and trends. According to the findings, both positive and neutral emotions accompany the public’s attitude toward digital fashion. There is a wide range of topics covered in the discussion. Innovations in digital technology have impacted the creation of jobs, talent demand, marketing strategies, profit forms, and industrial chain innovation of fashion-related businesses. Researchers in related fields will find this study useful not only as a reference for research methods and directions, but also as a source of references for research methodology. A case study and data reference will also be provided to industry practitioners.

## Introduction

Fashion is experiencing an accelerated digital transformation post-epidemic (LYSTinsight., 2021). Brands and designers appear to be undertaking some digital transformation between spring/summer 2020 and 2022, primarily by introducing cross-border games, 3D fashion, virtual models, online fashion weeks, etc. (Ramadan and Nsouli, 2022). Digital transformation has affected all aspects of fashion. For example, it has contributed to a shift in fashion communication and marketing models, with most companies and brands adapting their businesses through this industry-specific approach (Noris et al., 2021). It has also affected culture and society, influencing education and changes in design approaches and forms of artistic creation (Ryan, 2020; Zhao et al., 2021).

When there is the introduction of industry shifts or new products, fashion companies, brands and designers usually assess the impact of brand and market trends through the review of consumers’ feedback, desires and concerns (Bozhuk et al., 2019). This view conforms to Dhaoui’s (2014) research conducted earlier. According to the research, consumers’ feedback, opinions and comments are so important resources that they can provide a brand or enterprise with assistance in their response to the market quickly, prediction of the market potential or development direction on such basis, and then make timely adjustments to marketing strategies and the design of strategies (2014). However, previous studies on consumer feedback or sentiment have traditionally collected or analyzed data within enterprises using certain survey methods, such as focus groups or observation (Kendall, 2014). However, significant limitations are also available in this method, such as issues about skewed results due to the small sample size that cannot be employed in a comprehensive analysis.

Accelerated by the advancement in science and technology, social networks have changed people’s lives to a significant extent. Online social networks (hereinafter referred to as “social networks”) have four characteristics, namely, swiftness, communication, equality and self-organization (Borgatti et al., 2018). It is due to these characteristics that social networks have been employed by billions of users in just a few decades since the advent of the Internet, and have imposed its great impact on every field of our society. Moreover, social networks have also been described as the best platform for the exploration of the world (Ribeiro-Navarrete et al., 2021). The discussion of a particular topic or event usually promotes the formation of a specific virtual community in a social network and the generation of discussion and text data in a large amount. Named as user-generated content of social network, these data are namely user-generated content (Saura et al., 2022). Krumm et al. (2008) held the view that user-generated content on social networks can be applied to the identification of the public’s cognition of an event, phenomenon, topic, etc., so as to discuss the development status and create new content or knowledge on such basis (2008). This view is also confirmed by the relevant research conducted by Krippendorff (2018) and Eger et al. (2021). Krippendorff (2018) proposes the statement that understanding individual feedback and collective cognition in the community can offer strong support to information retrieval, information recommendation, information dissemination control and public event control (2018). It is critical to understand the characteristics of interaction and relationship which influence page visitors’ quick response to their target customers and prospects (Eger et al., 2021).

Based on the research mentioned above, this paper collects the popular user-generated content for digital fashion and virtual fashion, followed by the deduction of the development status shown by digital fashion, radiation field and future development trend.

This study designed several research questions to achieve the research goal as follows.

RO1: the widely used parts in the fashion field;
RO2: The impact of digital transformation and technology development on the fashion industry or society;
RO3: The views and attitudes toward digital fashion held by the public.

In terms of research methodology, we have assembled some computer algorithms to do data collection and analysis, including social network analysis (Valeri and Baggio, 2021) sentiment analysis (Arora et al., 2021) and subject analysis (Garcia and Berton, 2021). In text mining, topic modeling is one of the most powerful techniques used for data mining, discovering potential data sources, and determining the relationship between data and text. Latent Dirichlet Allocation (LDA) is one of the most widely used methods for topic modeling (Jelodar et al., 2019).

This study will analyze the content of relevant posts on Twitter, collect data using Python technology, and analyze relevant content using three text-based big data analysis methods, including topic modeling, sentiment analysis, and semantic network construction, in order to uncover deep feelings within the content. To achieve these objectives, our research consists of three steps:

(1) High-frequency keywords are identified using topic modeling and K-means clustering analysis;
(2) An analysis of sentiment is used to determine the emotional cognition and psychological characteristics of the public;
(3) Using co-occurrence matrixes and LDA topic modeling, network semantics are constructed, and key topics of social network content are identified.

In section “Literature review,” this paper reviews relevant literature. Section “Research methodology” focuses on the description of the methods. Results are displayed in Section “Results and analysis.” Section “Discussions” offers the results and discussions of open-ended innovation and a roadmap for future research. Finally, section “Conclusion” proposes conclusions, theoretical and practical implications, and the limitations at present.

The innovation of this research lies in the application of the technology related to data mining in the emerging topic of “digital fashion.” As an emerging field, there are few studies on the topic and it is even rare to see user-centered research. This study attaches importance to digital fashion and investigate users attitudes and emotional cognition toward emerging art styles and industrial changes using the method of information technology. As a result, it further expands the boundary of current research related to fashion topics. From the standpoint of public concerns, discussion points, and cognition, this study investigates the current state of digital/virtual fashion transmission. A combination of quantitative and qualitative research methods is employed in the present study, along with computer quantitative methods for processing textual perceptions in order to identify the current trends in digital fashion and virtual fashion, as well as the underlying context derived from public perceptions and emotional characteristics. This book provides participants and researchers in the fashion industry with theoretical and practical references.

## Literature review

As a result of digitalizing the fashion industry, it was hoped to streamline the design, production, and business of physical products for the real world, as well as to achieve sustainability through the use of various digital tools (Sayem, 2022). Its emergence is primarily dependent on “Industry 4.0.” Digital technology advancements, including network physical space development, the Internet of Things, computing tools, and digitization of fashion heritage, indicate the fashion industry is transitioning into the digital world (Kalbaska et al., 2018; Nobile and Kalbaska, 2020; Noris et al., 2020). Noris et al. (2021) divide the existing research on digital fashion into three major areas.

(1) Changes in Communication, marketing, and email marketing. Email information push services in digital format remain an important and widely used form of communication, based on the findings of the study. The three critical aspects of email marketing are cognition, emotion, and finance (Nobile and Cantoni, 2021). Text analysis was mainly used in this research, and then Nvivo software was used to quantitatively analyze the text content.
(2) Changes in business models. Berman (2012) put forward the statement that in the fashion industry, circular business models based on digital technology are composed of blockchain supply chain model, service innovation model and demand-driven model. This study lays emphasis on the methods of text analysis and review research. Apart from incremental innovation, aggressive business models including demand-driven models and digital innovation may be of great significance to the transformation of a circular economy in the field of fashion. The demand-driven model has the potential to shift the economy from scale to scope, changing the entire process from forecasting and production to the use of fashion goods and changing consumer behavior (Casciani et al., 2022). This study used a method of qualitative analysis with the aim of extracting high-frequency words from the text, followed by the summary of the content.
(3) Changes in design methods and creation methods. This field consists of 3D virtual simulation systems (Sina and Wu, 2022), collaborative design and customization of online platforms, and the prospects for 3D virtual clothing in the fashion and gaming industries (Donatiello et al., 2018). Research of this kind mostly uses the research method of experimental test to verify the possibility of the technology. The topics related to digital technology, dynamic range, wearability, expressiveness, interactivity and sustainability have triggered the discussions on possible digital fashion designs in the future (Choi, 2022).
(4) Changes in employment demand. The fashion industry, for example, is experiencing a digital transformation that is being accelerated by the COVID-19 pandemic. With the development of digital fashions, the skills and competencies required by the industry employees will experience significant changes (Nobile et al., 2021). As designers and creatives reshape the future of fashion systems in the digital world and digital fashion, there is the existence of the barriers to diversity, equity, and inclusion. However, there will be more job opportunities because of the greater equality in digital fashion and Web3 (it is the idea of the World Wide Web of a new version leveraging blockchain technology) (Sefidabi, 2022). Most of these studies adopt the research method of literature review.

The quantity of studies in the field of digital fashion research has widened, but most of them focus on the aspects covering the development status and prospects of the industry. There are very few studies related to the preferences of the public or consumers, perceptions, and perspectives, and even fewer relevant studies which extend from the public perspective to the prediction about the trends across the industry. Therefore, the combination of micro and macro features with consumers’ cognition-driven approaches can provide research with many different insights and new theories.

The corresponding research concept is the path of research development centered on public data that enriches the research paradigm in the business field (Lies, 2019), and the data and information obtained based on user evaluations is called user-generated data (UGD) (Saura et al., 2021). UGD includes all forms of information and data that are individually generated by users through their interactions with the elements that make up any digital marketplace (behaviors, experiences, feelings, reviews, comments, etc.) (Saura, 2020). The relevant research methods including natural language processing, text analysis, and computational linguistics, text mining can identify and extract information from unstructured text data, such as posts and user comments (Kim and Kim, 2014). As part of text mining, natural language processing and morphological analysis are used to determine the frequency and probability of occurrence of words in order to determine the relationships between texts (Callon et al., 1983; Srivastava and Sahami, 2009). This is a form of machine learning that employs a variety of analytical techniques, such as word class, degree centrality, and occurrence frequency analysis (Feldman and Sanger, 2007). As a result, it can provide historical data for the analysis of social networks or sentiments (Choi and Lee, 2020; Lang et al., 2020). Using natural language processing, text analysis, and computational linguistics, text mining can identify and extract information from unstructured text data, such as posts and user comments (Kim and Kim, 2014). As part of text mining, natural language processing and morphological analysis are used to determine the frequency and probability of occurrence of words in order to determine the relationships between texts (Callon et al., 1983; Srivastava and Sahami, 2009). This is a form of machine learning that employs a variety of analytical techniques, such as word class, degree centrality, and occurrence frequency analysis (Feldman and Sanger, 2007). As a result, it can provide historical data for the analysis of social networks or sentiments (Choi and Lee, 2020; Lang et al., 2020).

There is some research about use of computer algorithms for text mining user-generated content, and use this way to forecast the market trend prediction. Table 1 showed some relevant research.

**TABLE 1:** Existing relevant studies in fashion field.

Description	Methods	Authors
Analyzing stakeholders in the fashion industry, designing or testing products, and making changes to digital marketing strategies	Data mining-based topic modeling	Choi et al., 2021
The public response to the four global fashion weeks and the fashion trends revealed by them	Text data analysis	Choi et al., 2021
Audience perception mining using computer technology was conducted to investigate the impact of COVID-19 on the fashion industry	Structural equation modeling (SEM) techniques	Vãtãmãnescu et al., 2021
Consumer experience with online fashion rentals	LDA topic modeling and a co-occurrence network	Lang et al., 2020
Customer data analysis and can assist companies or brands in determining customer buying tendencies and preferences based on objective data	Artificial neural network algorithms	Nica et al., 2022a
Demonstrate consumers’ perceptions of gender-less fashion	Text data analysis	Kim et al., 2022
Customer brand perception and satisfaction	AMSTAR, Dedoose, Distiller SR, and SRDR.	Kliestik et al., 2022
Online purchase decision-making algorithms	AMSTAR, Distiller SR, ROBIS, and SRDR.	Hopkins, 2022

According to the findings of the preceding study, the computer algorithm technique is an effective method for extracting potential information and cognition by combining a variety of topics. However, little research has been conducted on digital fashion and virtual fashion. However, there is still a gap between actual social needs and academic research when it is used. Through the application of artificial intelligence and computer algorithm techniques to online data collection and data analysis, this study provides a comprehensive understanding of audiences’ actual perceptions of digital fashion and virtual fashion. The study uses both quantitative and qualitative methods, combining different ways of analyzing the data and providing new insights into the discussion.

## Research methodology

### Research design

Specifically, this study aims to investigate the public’s views and attitudes related to “digital fashion” and “virtual fashion,” as well as the hot topics discussed by the public.

Research question 1: In terms of digital and virtual fashion, what posts are the most popular at the moment?

In order to collect relevant posts and comments from Twitter, the researchers used the Twitter Application Programming Interface (API), searching for the keywords “virtual fashion” and “digital fashion”.

Research question 2: Which topics and keywords are hotly debated by the audience in the fields of digital fashion and virtual fashion?

Topics and keywords are identified using k-means clustering analysis and topic modeling techniques. Fashion’s current state of development in digital and virtual media can be viewed from a variety of perspectives and analyzed in depth.

Research Question 3: What is the public sentiment toward digital fashion and fashion in general?

Data mining is conducted using sentiment analysis methods.

An analysis of posts on social networking sites, such as Twitter, about digital fashion and virtual fashion was conducted. The Twitter service is a microblogging service that allows users to send short messages (tweets) that cannot exceed 280 characters and may contain images, videos, or hyperlinks to other websites. It is possible for users to retweet tweets (retweets) to their web-based networks or communities (followers) (Power, 2015). As a result of its global popularity and ubiquity, Twitter is a fast, accessible, and cost-effective medium for information communication and sharing (Erskine and Hendricks, 2021).

**FIGURE 1:**
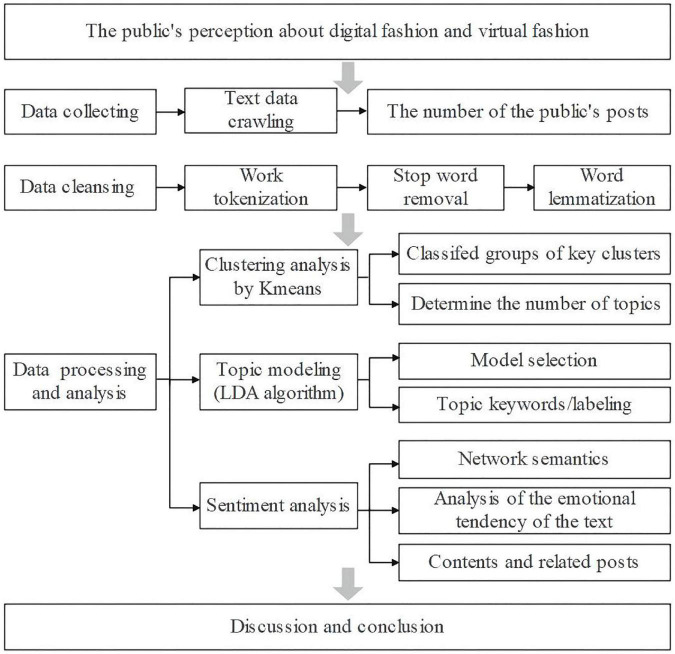
Research mythology.

Data was collected using Python technology and processed using cluster analysis, topic modeling, and sentiment analysis. As shown in Figure 1, the specific research methods and the research flow chart are described. The specific research steps consist of three parts:

(1) Data collection. I used Python 3.7 to collect public posts that contain the keywords “digital fashion” and “virtual fashion.”
(2) Data purification. In a broad sense, data cleaning includes a number of operations, including the capture, extraction, collection, screening, addition, deletion, modification, and reorganization of data. A data cleaning process refers to the process of removing invalid and incorrect data and leaving clean data, which involves the deletion, addition, decomposition, and reorganization of original data. In other words, it is the process of eliminating redundancy, noise, errors, and inconsistencies. Following the collection of text data and analysis of the collected data, the next step is to answer the research questions outlined above, which is the stage of data processing and analysis. There are three steps in this section.

In the first instance, this research will use K-means to identify and classify the text data in order to determine the number of topics based on the huge amount of information available. This step is helpful for the classification of information and for finding the main information since posts are collected based on two keywords. Then, using LDA, find the keywords based on the topic and label the data to generate a word cloud. The purpose of this step is to reveal to the general public the main contents of digital fashion and virtual fashion. As a result, sentiment analysis was adopted. Analyzing the emotional tendency in text data and exploring the public’s response and sentiment with regard to this phenomenon using network semantics. Data mining is conducted using sentiment analysis methods. In sentiment analysis, key opinions, emotions, attitudes, and tendencies are extracted from large text datasets in order to estimate and categorize the author’s emotions (Feldman, 2013; Lee et al., 2016). Words with an emotional orientation are also known as polar words and evaluation words (Ghiassi et al., 2013). As of now, sentiment analysis is dominated by methods based on the construction of sentiment dictionaries and machine learning techniques. It examines the text’s words using an emotion dictionary, calculates the emotion value, and then evaluates the emotion value to determine the text’s emotional disposition (Oh and Chae, 2015). To find out what people think and feel about virtual fashion and digital fashion, the research is conducting a survey. While the data is displayed, explain and demonstrate the results of data analysis combined with existing research. The research will discuss and summarize the final results after a series of analyses.

### Research methods of data collection and analysis

The post text content was analyzed and refined using four different research methods, including Co-Occurrence Matrix, LDA Topic analysis, K-means clustering analysis, and Sentiment Analysis.

#### Co-occurrence matrix

In cluster analysis, research objects are classified automatically according to their characteristics using data reflecting those characteristics without the need to define the classification criteria (Jia, 2010). Three characteristics of cluster analysis can be summarized: clustering is an effective method for classifying rather than creating a unique classification; in terms of the analysis object, clustering deals with two-dimensional tables in the form of properties-records; in terms of the application prospect, clustering is an efficient method of unsupervised classification which will become increasingly popular in big data environments. Using cluster analysis, the current research analyzes different types of co-occurrence matrices to automatically extract meaningful topics from data of a certain size (Zhou et al., 2014).

A process for generating co-occurrence matrixes (Thelwall, 2001; Bar-Ilan, 2006).

1. Co-occurrences of words in the corpus are stored in matrix A, which is formed by all the non-repeating words in the corpus.
2. It is possible to artificially specify the size of the Context Window, and then count the number of times each word appears in the specified size of the Context Window along with the surrounding words.
3. Counting the number of times each word pair occurs in the corpus.

#### Latent Dirichlet Allocation topic analysis

A topic model was developed based on the Latent Semantic Indexing (LSI) approach proposed by Papadimitriou et al. (2000) It is the basic idea of LSI to construct a new latent semantic space with a lower dimensionality than the original space through singular value decomposition (SVD), so as to find a simpler representation of the text (2000). Hofmann (1999) also proposed probabilistic LSI based on LSI word classification (pLSI), which differs from LSI by taking the optimal low-rank approximation as the optimization goal, whereas LSI takes the likelihood value of observations as the optimization goal and maximizes it. Based on Bayesian theory, (Blei et al., 2003) created LDA in 2003, which considers the parameters to be evaluated in probabilistic LSI as random variables with Dirichlet prior distributions. By inferring the underlying content variables, the LDA algorithm aims to model a corpus. We may consider these papers to be content-related groups or categories (Ming et al., 2022).

**FIGURE 2:**
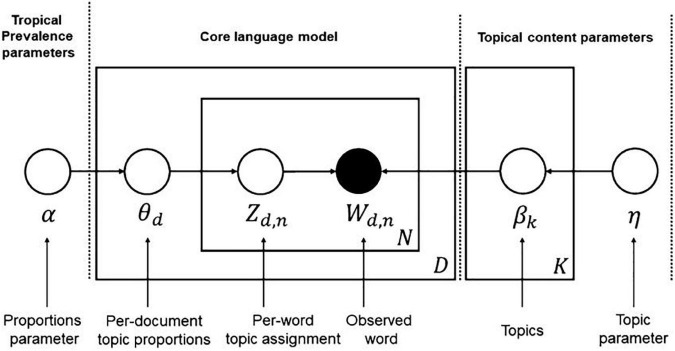
The LDA model from Blei (2012, p. 81).

As shown in Figure 2, Blei (2012) explained the LDA model algorithm as follows: the square boxes are called “plates,” and “N” is a collection of words within a document, “D” is a collection of documents, and “K” is a set of topics. The circles represent probability parameters, and the node in, ncircles represas a word in the document; while topics, topic distributions, and topic assignments are not revealed. There are full words (“Wd, n”) in the numerous documents (“D”) collected by the researchers, assuming that each word corresponds to a topic (“Zd,n”) (Kim et al., 2022).

#### Latent Dirichlet Allocation topic analysis

By learning from unlabeled samples, cluster analysis aims to discover hidden patterns, subsets, and correlations within a data set, thus allowing unlabeled data objects in the data set to be classified into corresponding classes, called class clusters. By clustering, a reliable cluster analysis cluster partitioning method should make the partitioning result as similar as possible between data objects within the same class cluster (Xiao et al., 2022). In contrast, it should be as different as possible between data objects within different class clusters.

The K-means clustering algorithm is the most fundamental and widely used clustering algorithm. Its basic concept is to find iteratively a classification scheme for K clusters that minimizes the loss function corresponding to the clustering results. The loss function can be defined as the sum of the squares of each sample’s error from the cluster’s center (Likas et al., 2003):


$$J\left(c,/m\mu \right)=\sum_{i=1}^{M}||x_{i}-mu_{c_{i}}|{|}^{2}$$

Where *x_i_* represents the it samples, represents the cluster to which belongs, /*mu*_c_i__ represents the center point corresponding to the cluster, and *M* represents the total number of samples.

Steps involved in the operation:

1. Select K points at random from the sample as the initial centroid;
2. Divide the samples into clusters corresponding to the nearest centroid by calculating the distance between each sample and each centroid.
3. Calculate the mean value of each cluster’s samples and update the cluster’s center of mass with the mean value.
4. Steps 2 and 3 should be repeated until one of the following conditions is met: The maximum number of iterations is reached when the change in the centroid position is less than the threshold value (default is 0.0001).

#### Sentiment analysis

In sentiment analysis, key opinions, emotions, attitudes, and tendencies are extracted from massive texts in order to estimate and classify the author’s feelings (Feldman, 2013; Lee et al., 2016). The study used the SentiWordNet Emotion Dictionary, a semantically rich English dictionary developed and maintained by the Cognitive Science Lab at Princeton University under the direction of psychology professor George A. Miller. In WordNet, the terms are categorized by their meaning, and each group of terms with the same meaning is referred to as a subset.

Next, they are loaded into a Python programmer equipped with the Naive Bayes Analyzer, and a tagged Digital fashion/Virtual fashion corpus serves as the training set. As part of this algorithm, all comments are considered subjective and are assigned a positive and negative value with a sum of 1.

Comments that have a positive evaluation/emotion value greater than 0.5 are considered as positive evaluations/emotions. The comment is determined to be negative evaluation/emotion dominant if the negative evaluation/emotion value exceeds 0.5. When the positive and negative evaluation values are equal, the comment is considered neutral (Zhang, 2019).

## Results and analysis

The Python program used the open Twitter API to search for relevant posts and comments on such topics using the keywords “virtual fashion” and “digital fashion,” as well as the date, title, content, likes, comments, re-tweets, and link information of these posts. Following the preprocessing, special characters and punctuation marks were removed from the data. A total of 528,915 records were collected between October 2020 and May 2022. For the data preprocessing, the Python3.7 natural language toolkit (NLTK) was used, and the model was constructed using the Web crawler language. Public posts about “digital fashion” and “virtual fashion” were collected. The posts were sorted by the number of likes using the keywords “virtual fashion” and “digital fashion.” The higher the number, the greater the audience recognition and the greater the attention paid to it. According to the number of likes, a portion of the collected metadata is sorted as follows, shown in Table 2.

**TABLE 2a:** Original posts collected by keyword “digital fashion” on Twitter.

User name	Issuing time	Number of likes	Number of comments	Forwarding number	Contents
Gastt_fashion	2022-03-21 18:30:18	296,402	918	38,252	Footwear design by Davina India. https://t.co/K1EJzw3bNl
Gastt_fashion	2022-03-21 18:30:18	296,402	918	38,252	Footwear design by Davina India. https://t.co/K1EJzw3bNl
Delorestaurus	2021-07-25 00:34:32	225,881	396	25,576	After the era of malls ends eventually someone will say what if we shopped online, but in real life? And then malls will be reinvented
Ukraine	2022-02-26 15:29:09	214,482	11,384	57,959	Stand with the people of Ukraine. Now accepting cryptocurrency donations. Bitcoin, Ethereum and USDT.
Aespa_official	2021-09-25 15:00:03	199,889	4,080	90,482	Aespa 에스파〖Savage〗싱 https://t.co/dTlcVZuNge
Aespa_official	2021-09-25 15:00:02	191,759	3,782	84,367	Aespa 에스파〖Savage〗싱 #Savage #싱크다이브 #SYNKDIVE https://t.co/EKruWVS31X
Aespa_official	2021-09-25 15:00:02	191,759	3,782	84,367	Aespa 에스파〖Savage〗싱크다이브 #Savage #싱크다이브 #SYNKDIVE https://t.co/EKruWVS31X
Aespa_official	2021-09-25 15:00:06	176,454	2,877	77,308	Aespa 에스파〖Savage〗싱크다이브 #WINTER #윈터 https://t.co/GRCRofAHwt
Aespa_official	2021-09-25 15:00:08	173,798	3,163	74,748	Aespa 에스파〖Savage〗싱크다이브 #Savage #싱크다이브 #SYNKDIVE https://t.co/CftkF2lDEN

**TABLE 2b:** Original posts collected by keyword “virtual fashion” on Twitter.

User name	Issuing time	Number of likes	Number of comments	Forwarding number	Contents
BTS_twt	2021-01-21 12:39:18	2,531,067	129,587	588,114	https://t.co/yCKO3PZTIx
LouisVuitton	2021-07-07 10:00:01	1,202,676	11,868	310,589	Louis Vuitton presents the men’s fall-winter 2021 collection by Virgil Abloh in Seoul featuring house ambassadors BTS. https://t.co/tsAokz5l1d
Louis_Tomlinson	2020-12-07 18:03:53	498,052	80,560	60,460	Hope everyone is doing good! Excited for the show!
Yourubamagician	2021-02-24 00:11:08	486,576	3,989	113,894	Yo who let their dad on tiktok???? https://t.co/JWjajnSJjj
Elonmusk	2021-04-09 22:24:01	330,820	5,103	24,733	It’s all about the cufflinks https://t.co/elccqC0Zuf
Silkenhearts	2021-06-13 06:16:38	224,227	330	33,774	The original it girls https://t.co/yFiiS891Za
Aespa_official	2021-09-28 15:00:09	165,730	3,496	73,611	Aespa 에스파〖Savage〗환각 #HallucinationQuest https://t.co/Bxp9a0Hk8w
Kourtinthakut	2021-01-07 18:07:00	158,241	313	29,251	Conversation pits, 1970 https://t.co/EoRWw8RQJY
Slickjit	2021-06-27 20:45:04	150,071	299	17,025	Sent him my outfit n he said cute who want me? like fr

Table 3 displays the high-frequency keywords that were extracted using topic modeling and K-means clustering analysis.

**TABLE 3:** Results of the word frequency analysis.

Rank	Word	Frequency	Rank	Word	Frequency
1	Fashion	46,071	16	Design	1,884
2	Virtual	24,043	17	Brand	1,883
3	Digital	21,811	18	Art	1,683
4	Amp	7,253	19	Check	1,672
5	Metaverse	5,296	20	Real	1,635
6	Week	4,406	21	Designer	1,549
7	Nft	3,113	22	Time	1,482
8	Future	2,744	23	Global	1,480
9	Join	2,694	24	Designers	1,470
10	Industry	2,423	25	Live	1,454
11	Collection	2,420	26	People	1,423
12	Nfts	2,385	27	Physical	1,388
13	Brands	2,290	28	Online	1,383
14	Event	2,037	29	Day	1,319
15	Pm	1,906	30	Love	1,304

**FIGURE 3:**
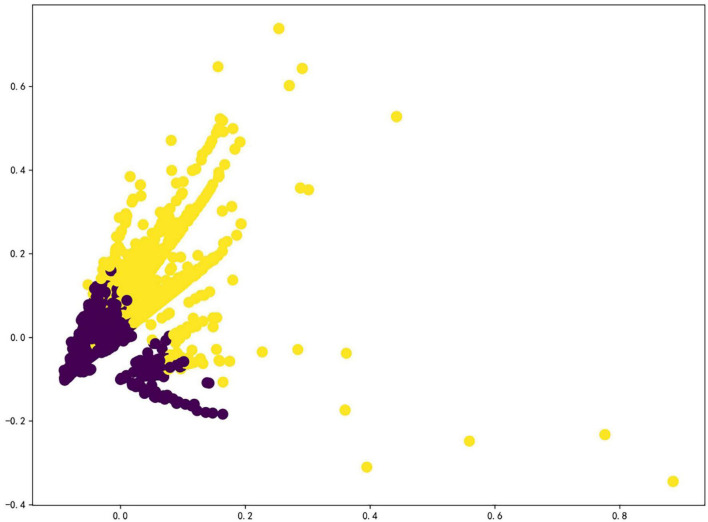
Visualization of cluster data.

Following the preprocessing of the data and the conversion of the word vectors, Figure 3 illustrates the clustering phase of the experiment.

As a general rule, contour coefficients range from –1 to 1, and the greater the value, the greater the distance between the cluster and other clusters, and the more compact the distance within the cluster. Furthermore, an excess or deficiency of data within a cluster may affect the persuasiveness and representativeness of the clustering results. Therefore, determining the *K* value requires a thorough evaluation of the contour coefficient value and the distribution of data in the scatter graph. The more uniform the distribution of class cluster data, the more reasonable the *K* value will be when the contour coefficient reaches its maximum value (Krishna and Murty, 1999).

As a result of word vector transformation and clustering, the original text data without any rules were divided into two classes in a reasonable and effective manner. The requirement topics covered in the question text generated by users are often diverse and personalized, and the class cluster contains numerous data points, making it difficult to identify the class cluster’s core characteristics directly; thus, LDA topic extraction is necessary.

**FIGURE 4:**
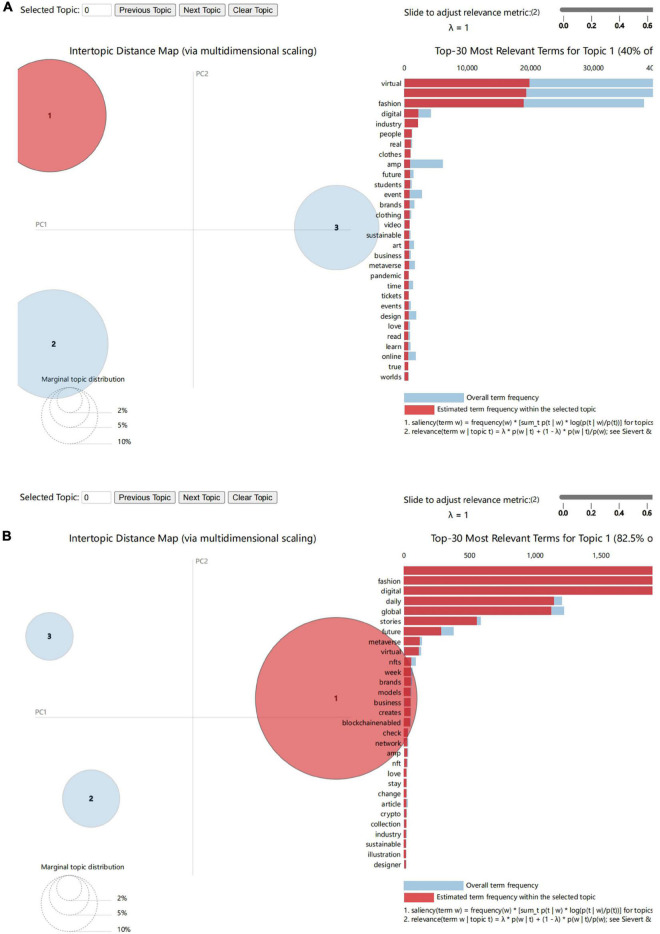
**(A)** Topic modeling for the first cluster class. **(B)** Topic modeling for the second cluster class.

The following steps are involved in extracting LDA topics from the entire dataset:

(1) Read the data, load the stop word table, preprocess the data, and create a tuple that consists of particle, word class tagging, stop word removal, word, and word class.
(2) The vectorization process involves transforming words into a matrix of word frequency.
(3) Extract keywords and convert them into vectors by conducting a statistical analysis of the TF-IDF weights of the words in the matrix.
(4) Determine the level of confusion, determine the optimal number of topics in LDA, define the function and output the keywords for each topic, and complete the topic keyword extraction.
(5) Visual analysis: present the results of the LDA topic analysis in a way that makes them interactive and dynamic. This step requires the use of the Potential Dirichlet Allocation (LDA) provided by the Gensim package. The first step is to import the packages. Among the core packages are re, gensim, spacy, and pyLDAvis. Additionally, matplotlib, numpy, and PAses are required for data processing and visualization. In addition to importing deactivated words from the NLT, some additional words were also added to the original list of deactivated words. It is necessary to clean up the dataset before starting topic modeling. Email links, extra spaces, and line breaks should be removed, each sentence should be marked as a word list, and punctuation and unnecessary characters should be removed. Build the bigram and trigram models, then define functions for stopwords, bigrams, trigrams and lemmatization, create the lexicon and corpus needed for topic modeling, and then enter the core step—LDA models are built for different topics, and each topic is a combination of keywords, and every keyword has a certain weightage in the topic. The Perplexity and Coherence Scores are calculated to determine the relevance and complexity of the generated topics. In order to visualize our model, the research recommend using pyLDAvis. The purpose of pyLDAvis is to assist users in interpreting topics in a topic model that fits a corpus of text data. The application extracts information from a fitted linear discriminant analysis topic model (LDA) in order to facilitate interactive web-based visualizations. A detailed analysis of the results can be found in Figure 4.

A variety of subjects are represented by circles. In each topic, the size of the circle corresponds to how much text is contained within it. An icon is depicted horizontally on the right which represents the 30 most relevant terms for the topic. It is evident that the two cluster classes are divided into three topics by the presence of three circles in each. The following 30 keywords correspond to the 30 most important keywords extracted from the entire text when the mouse is not over any particular topic. You will immediately notice a change in the list of keywords to the right when you hover over circles 1, 2, or 3. The data display for each circle turns red when the mouse is clicked, which indicates that the frequency with which each keyword appears in the current topic. The circles in the Figure will not overlap if the model fits well.

According to Figure 4, there is no overlap between the six topics. This indicates that there is little correlation between them and that each topic falls within a relatively distinct research area. Thus, after LDA topic modeling analysis, all data can be divided into 6 topics. By clicking on each topic circle, a bar graph with the top 30 most relevant terms for that topic will appear along with the keywords corresponding to it. Due to the fact that computer information technology only provides statistics, the content of each topic must be summarized using keywords. Based on an analysis of the lexical nature of the keywords, the six topics can be classified into the following categories, as shown in Table 4.

**TABLE 4:** Keywords in topic clustering.

Topics	Keywords
1.1 Era, group, and attitude involved in virtual fashion/digital fashion	Virtual, fashion, digital, industry, people, real, clothes, future, students, event, brands, clothing, video, sustainable art, business, metaverse, pandemic, time, events, design, love, read, learn, online, true, and worlds
1.2 It mainly reflect the fashion context involved in virtual fashion/digital fashion	Virtual, fashion, week, digital, design, reality, experience, party, online, metaverse, collection, showcase, designers, brand-runway, designer, brands-store, platform, style, New York, art, London, luxury, nft, shop, and app
1.3 It mainly refers to the time, occasion, and descriptive words mentioned in the post	pm, event, register, live, link, week, holiday, day, free, November, march, tomorrow, red, watch, time, Friday, carpet, tonight, style, amazing, night, annual, zoom, Saturday, and Instagram
2.1 The present situation and development trend of virtual fashion/digital fashion	Fashion, digital, daily, global, stories, future, meta verse, virtual, nfts, week, brands, models, business, creates, blockchain enabled, check, network, nft, love, stay, change, article, crypto, collection, industry, sustainable, illustration, and designer
2.2 Industry or content involved in virtual fashion/digital fashion	Digital, future, fashion, global, blockchain, nfts, daily, stories, read, luxury, report, sense, metaverse, investing, gaming, thinking, bright, summit, potential, article, amazing, industry, virtual, brands, wearing, surveyed, people, and nft
2.3 Impact of digital/virtual fashion on peoples life and the world	Daily, global, inside, international, virtual, future, stories, scenes, week, summit, investing, launches, clo, county, galveston, community, register, creators, speaking, news, tech, trends, emagazine, visualize, fabrics, relatable, and lives

Based on the combination of the six themes for analysis with current research, it is evident that the keywords covered under each theme are relevant to current industry topics. Through the introduction of VR, AR, and AI technologies, industrial innovation has been promoted (El-Sayed and El-Fanagely, 2022), as well as emerging research and industries around digital people, virtual people, and virtual beauty, which are being extended globally, such as virtual wear and NFT, with companies such as Auroboros, DressX, and Tribute Brand introducing virtual wear digital products. According to Morgan Stanley, a well-known investment bank in the United States, the market for virtual wearable products is expected to reach $56 billion by 2030 (Astakhova and Kalyazin, 2022). The concept of “bionics” is incorporated into some of the virtual wearable designs, adding a new dimension to the design of costumes in both virtual and real environments. It is not only a matter of breaking through the limits of traditional manufacturing techniques; it has also had a significant impact on retail. It is not only the development of virtual fashion that creates new economic models and industrial channels. However, it is also the result of the ongoing process of constructing and constructing human identities. While the incarnated image of the virtual world is based on the natural world of self-expression and value influence, virtual wear is the technological realization of expressing identity and self (Choi, 2022). The sustainability and fashion technology industry writer Brooke Roberts-Islam explained in a Forbes article entitled “Why People Want to Wear Digital Fashion” that all clothing impacts the environment and puts pressure on the planet’s ecology from a rational and environmental perspective. While some believe that the only truly sustainable solution is to go nude, digital fashion has become the next-best alternative. According to DressX, the world’s first multi-brand digital fashion retailer, producing a digital fashion item reduces carbon emissions by 97% compared with producing a physical item (Roberts-Islam, 2020). All six themes contain key words that are closely related to the above. Technology is bringing about the expansion of design boundaries, the importance of sustainability, the impact on industrial models changing, and the reflection on human thought.

**FIGURE 5:**
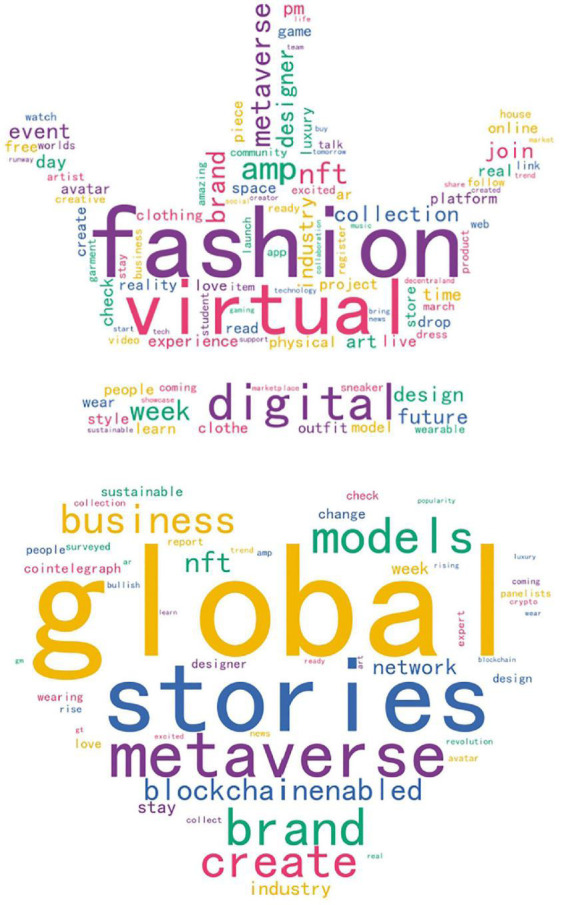
Clustering word cloud.

With the use of the wordcloud tool, high-frequency keywords are extracted from the data of each class cluster. The size of the word cloud will also increase if the frequency of a word is greater. The details are shown in Figure 5.

It is evident from Figure 4 that the two class clusters have obvious theme features, diverse core words, and a large number of lexical items, indicating that the public is clearly interested in the contents of the classes. Among the words associated with the first class are “fashion,” “virtual,” and “digital,” which are also the keywords of this study, followed by “amp,” “metaverse,” “week,” “NFT,” “brand,” and “collection.” The abbreviation “amp” refers to the nonsensical word ampersand. Currently, “metaverse” is the most popular topic, which is linked and created using technology. In the meantime, it is a digital living environment, incorporating a new social system and a virtual world that maps and interacts with the physical environment. This protocol identifies an ecologically indivisible and unique interface specification for Token interaction and circulation, enabling the marking of native digital asset ownership (i.e., assets that exist in the digital world or originated there). Its key innovation is its ability to mark native digital assets as belonging to their owners. As tokenized tickets and one-of-a-kind jewelry are non-homogeneous and non-disintegrable, they simultaneously ground the concept of real-world goods. It is possible to tokenize any valuable creation and track the ownership of the information using NFT, thereby realizing the intersection between information and value. It is the first time that the fashion industry has been in contact with NFT through the collaboration between digital artists Fevocious and RTFKT. In 2019, The Fabricant sold its first piece of digital haute couture for $9,500. Blockchain technology can be used to convert a fashion item into a legitimate investment. NFT is a novel marketing strategy for the digital fashion industry.

The high frequency of the word “brand” suggests that the general public is also discussing the fashion brands that are driving digital fashion, including OTB, Louis Vuitton, Gucci, Balenciaga, Dior, Ralph Lauren, Vans, Nike, Adidas, and so on, which are actively involved in the creation of digital fashion art and in the expansion of the digital fashion segmentation market. It is possible to demonstrate your capabilities by creating a metaverse department, releasing NFT products, creating virtual stores, and working with virtual idols.

Among the key terms used in the second group are “global,” “stories,” “metaverse,” “brand,” “create,” “business,” “models,” and “blockchain-enabled.” The concept of the metaverse has sparked a global wave of digital endeavors. Many nations around the world, represented by the fashion industry, have begun to produce and sell virtual clothing, virtual idols, virtual cosmetics, virtual accessories, and other works of art. As a result of the advent of digital fashion, the industry’s business model has evolved, as well as forecasting future operation models, market segments, and product innovations. In spite of the fact that time will determine whether digital fashion based on the metaverse concept is a bubble or a new blue ocean, it nevertheless offers a fresh perspective on the fashion industry. The purpose of the project is to establish a foothold in the virtual fashion industry, provide consumers with superior shopping experiences, especially for luxury brands, and develop digital fashion channels to enhance consumer interaction and communication.

Fashion is also concerned with sustainable fashion. Through virtual fashion, pollution caused by consumables such as clothing can be mitigated. An example is “blockchain-enabled” transacted energy (BCTE), which has the potential to create an open, trustworthy, and transparent energy market. In addition to reducing the cost of renewable energy investments, this will improve our ability to combat climate change, encourage more participants in the renewable energy sector, and boost innovation through transparency and grid access. Among the most commonly used words is “blockchain-enabled,” which indicates how much people care about the environment and resources.

All cluster types have independent and diverse topics within each cluster, which indicates that users’ information needs related to this topic are similar and cannot be fully satisfied. These clusters are not only dominated by nouns and lexical items but also have independent and diverse topics within each cluster. Therefore, this may reflect the public’s and consumers’ interest in and exploration of “digital fashion” and “virtual fashion.” In light of the influences of science and technology as well as the social environment, further investigation is necessary to determine if this new concept or product is capable of meeting the needs of the general public.

**FIGURE 6:**
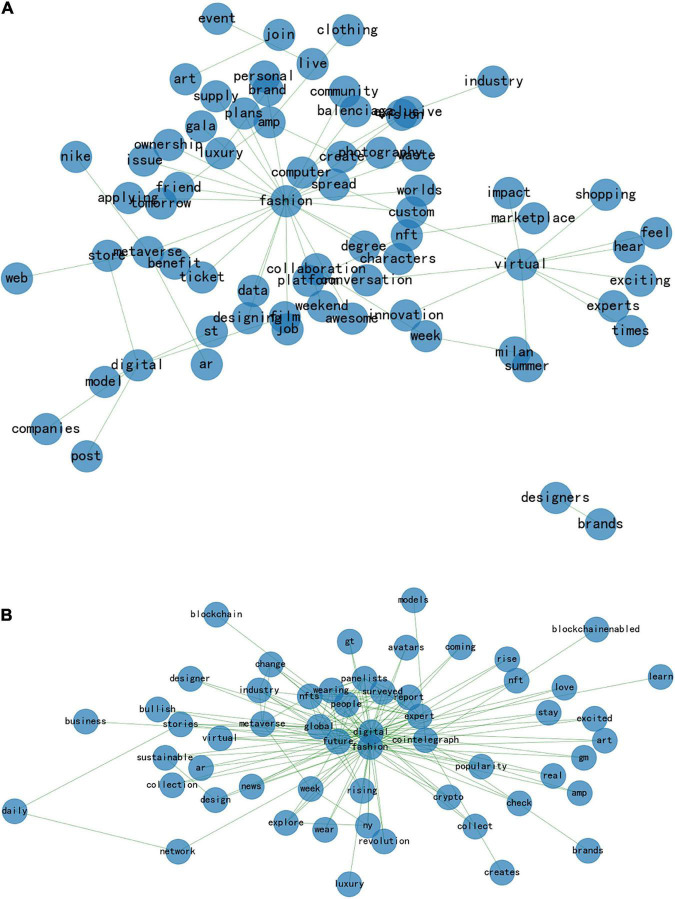
**(A)** Clustering—network semantics. **(B)** Clustering—network semantics.

It is noteworthy that LDA topic modeling and K-means cluster analysis can provide a preliminary understanding of the current hot topic of digital fashion for further investigation of their relationship. To analyze both the social network and the semantic network, a Python3.7 semantic analysis tool is utilized to analyze both the social network and the semantic network. Create a social semantic network structure map, display the structural relationship between words through graphical representations, analyze the correlation between text data sets based on user comments, and then mine the potential information from the data. Social semantic networks are illustrated in Figure 6.

According to Figure 6A, two major networks dominate the distribution of network semantics: fashion and virtual. Among these, “fashion” requires more categorization and explanation since it encompasses a broader and more dispersed range of activities. The network semantic diagram reveals that the words closest to the network center in the network with “fashion” as the core word are “computer” and “spread.” “Balenciaga” and “Nike” are categorized as brands. Due to Balenciaga’s presentation of video and video game depictions of the post-epidemic era from summer 2021 to fall 2021, the brand has been widely discussed. In the Spring 2022 runway show that debuted on June 6th, Creative Director Demna Gvasalia once again used fashion as a means of examining and interpreting the present while exploring the future. On social media platforms, this sparked a heated debate and attracted the attention of the general public worldwide. By joining forces with RTFKT, a digital fashion and footwear brand the company acquired last year, Nike demonstrated for the first time that it is selling sneakers in the metaverse (virtual space). With the help of NFT (non-homogeneous token) technology, Nike opened a store on Roblox, a popular online game with 50 million daily visitors, in a virtual sneaker market, promoting its brand in the virtual space and building relationships with young digital natives. Examples of successful digital fashion brands include Balenciaga and Nike.

Fashion includes clothing, design, and modeling. As a result of the epidemic, virtual fashion actively seeks to combine virtual technology and the fashion industry, as well as being influenced by the development of avatars and virtual platforms in the metaverse (Hollensen et al., 2022). Software and technology are used in a virtual fashion as opposed to an actual fashion. As a result, this change also led to changes in clothing shapes, design methods, design tools, and creative ideas in the fashion industry. For example, virtual fittings, virtual models, and virtual idols are all examples of how technology and creativity have changed over time.

It is common to use words such as “personal,” “create,” “awesome,” “innovation,” “exclusive,” “exciting,” “characters,” and “collaboration” to describe digital fashion. Generally, people feel and think about virtual fashion in more positive terms, as evidenced by the word classes of these descriptors.

“Community,” “Industry,” “Event,” “Platform,” “Worlds,” “Milan,” “Market,” “Place,” and “tomorrow” fall under the category of site and scene. During Milan Fashion Week, a total of eight digital shows were held between February 22 and February 28, 2022. In autumn/winter 2022, ANNAKIKI debuted its NFT virtual clothing capsule collection, introduced the concept of man-machine symbiosis, and introduced the concept of the “post-human era,” sparking heated debates on the Internet. The growth of digital fashion creates more “events” and needs more “platforms” on top of making the fashion industry more innovative.

The verbal terms include “vision,” “impact,” “hear,” “feel,” and “supply.” As an example, using the digital fashion show as an example, technologies such as interactive lighting, sound arrangements, and touch interaction are utilized to enhance the exhibition experience from traditional “watching” exhibits to multi-sensory stimulation of “vision,” “hear,” and “touch.” As a result, exhibits are no longer viewed as single objects but as a space where the real and the virtual interact, allowing the audience to be immersed in the artist’s world. Virtual and digital fashion have evolved inextricably linked to advances in science and technology, as well as changes in economic conditions.

Second, there is only one network center, and the center words are “digital” and “fashion.” Since “surveyed” and “reported” belong to the same word class, digital fashion is a popular topic across multiple industries. There are also three types of group words in the network semantic map, namely “Panelist,” “People,” and “Expert.” It suggests that the development of digital fashion has spawned relevant research groups, industry personnel, and their particular target consumers, as well as addressing the talent needs and stakeholder needs of each major stage of the entire industrial supply chain: digital brand operation, digital design and development, and digital brand management.

There are two additional terms associated with this network, namely “Coin Telegraph” and “NFTs,” which demonstrate its innovative nature. It has been the focus of the cryptocurrency industry since the second half of 2021 to develop Web3 and metaverse economies based on user-owned digital assets like NFT. While the global economy is currently experiencing a downward trend and the crypto market is experiencing significant losses, institutional investors, companies, and new crypto projects continue to focus on the development of innovative metaverse applications. There are a number of well-known brands that have begun to establish their presence in these new virtual worlds, including Nike, Gucci, Balenciaga, and Prada (Karamalak et al., 2021).

**FIGURE 7:**
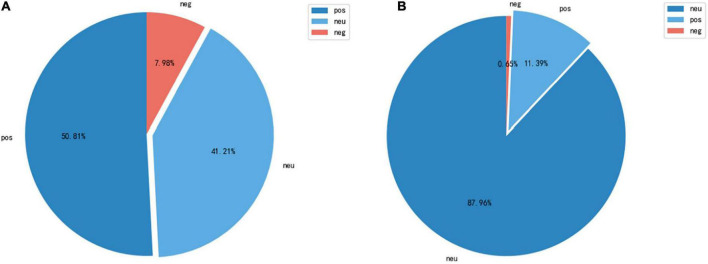
Sentiment analysis. **(A)** Cluster 1 sentiment analysis. **(B)** Cluster 2 sentiment analysis.

In Figure 7A, the positive attitude accounted for 51.81 percent of sentimental topics in the first category, the neutral attitude for 41.25 percent, and the negative attitude for 7.98 percent. As summarized in the first cluster of descriptive words, the era and attitude of the group as well as the fashion context involved in digital fashion, as well as the time, occasion, and descriptive words used in posts, are the primary focus.

There is no doubt that the majority of audiences do not resist digital fashion, and the majority holds a neutral attitude and is active in catering.

Based on the analysis of the descriptive words in cluster 2, the main content of cluster 2 includes the development trend of virtual fashion or digital fashion, the industry or content involved in virtual fashion or digital fashion, as well as its impact on the global community and people’s lives. In regards to the percentage of emotions associated with cluster 2, Figure 7B differs from Figure 7A, showing that a neutral attitude accounts for 87.96%, a positive attitude accounts for 11.39%, and a negative attitude account for only 0.65%. As a result of combining the cluster contents and emotion percentage data, it is evident that the public’s perception of this area is generally positive (The development of technologies such as virtual reality, intelligence design tools and processes, etc.).

Based on the reports from around the world, this perspective can be summarized as well. Consumers or advertisers may make these posts. In spite of the fact that virtual technology can enhance product marketing and consumer communication, most consumers still consider product material, technology, brand cultural connotations, and public identity before purchasing fashion items. Because of this, the majority of consumers remain wary of virtual digital fashion and the metaverse.

A number of brands, particularly luxury brands, take a wait-and-see approach: “Hermes is in a wait-and-see phase, and we use it to communicate with young consumers, but we are not in a hurry to enter the virtual world,” said Axel Dumas (CEO of Hermes) when asked about the recent popularity of metaverse topics.” As a result, we continue to spend 15 h on each bag.

The CEO of LVMH, Bernard Arnault, issued a warning against a metasomatic bubble at the beginning of this year, emphasizing that his company would not sell virtual shoes. As he stated, “We have no interest in selling virtual shoes for a price of ten euros.”

According to Table 5, there are a variety of opinions and feelings regarding digital fashion and virtual fashion.

**TABLE 5a:** Analysis results of neutral attitude and content of related posts.

Comments	Scores	Compound	Neutral
Power data digital fashion design	“neu”: 1.0	0	1
Flexi remote UK based email cv amp folio event	“neu”: 1.0	0	1
Metaverse reviving art fashion illustration	“neu”: 1.0	0	1
Global digital fashion daily stories	“neu”: 1.0	0	1
Power data digital fashion design	“neu”: 1.0	0	1
Digital fashion menu week dcl fashion voting digital fashion digest digital alchemist series hosted fabricant label preview nft berlin	“neu”: 1.0	0	1
Happening fashion industry answer learn	“neu”: 1.0	0	1
Emerging metaverse spaces digital real estate developer agent digital fashion designer usable games virtual event manager normalized people shifting	“neu”: 1.0	0	1
Caco designer	“neu”: 1.0	0	1
Global digital fashion daily stories	“neu”: 1.0	0	1

**TABLE 5b:** Analysis results of negative attitude and content of related posts.

Comments	Scores	Compound	Negative
Scared	“neg”: 1.0	–0.4404	1
Wrong	“neg”: 1.0	–0.4767	1
Fuck fuck fuck digital fashion fucking retarded fuck fuck fuck	“neg”: 0.893	–0.9776	0.893
Falling hell mind	“neg”: 0.861	–0.7351	0.861
Fud fashion fear	“neg”: 0.841	–0.6486	0.841
Fuck kill game sneaky access	“neg”: 0.835	–0.8779	0.835
Rstlss discord missing	“neg”: 0.831	–0.5994	0.831
Hell window	“neg”: 0.821	–0.6808	0.821
Killed sold	“neg”: 0.818	–0.6705	0.818

**TABLE 5c:** Analysis results of positive attitude and content of related posts.

Comments	Scores	Compound	Positive
Love love	“pos”: 1.0	0.8555	1
Fascinates creative fun	“pos”: 1.0	0.8481	1
Beautiful wow	“pos”: 1.0	0.8271	1
Hope win	“pos”: 1.0	0.7717	1
Gn sweet dream	“pos”: 1.0	0.7351	1
Supporting friend	“pos”: 1.0	0.7269	1
Congrats innovation	“pos”: 1.0	0.7184	1
Wonderful creation	“pos”: 1.0	0.7003	1
Dear engagement	“pos”: 1.0,	0.6808	1

According to a sentiment analysis of textual data, the public’s attitude toward digital fashion is predominantly positive or neutral. In contrast to traditional fashion, digital fashion emphasizes artistic creation while having its own value.

Environmental Value: In the traditional fashion industry, there has always been a problem of “overcapacity,” and virtual fashion, which uses only digital images without physical carriers, has become a new perspective in regards to industrial pollution under the heading of “sustainable fashion.”

Social Value: In addition to satisfying people’s imaginations of clothing, virtual fashion artwork can also convey personal emotions and psychological characteristics by using bold colors and exaggerated silhouettes since it is not limited by the physical carrier. Virtual fashion is consumed by the public and shared on social media or the Internet to demonstrate their own distinctive aesthetics and desire to explore the future.

Arts Creation and Imagination Value: In spite of the fact that the human imagination is limitless, the physical carrying of entities is subject to a variety of factors. Virtual fashion can meet the creative needs of various art forms and express emotions or concepts in a variety of ways.

Tolerance Value: You can obtain a piece of clothing virtually, especially for those with special needs, such as the disabled. The combination of a virtual image and virtual clothing is capable of removing the actual physical limitations.

As a result of these characteristics, some groups have a positive attitude toward digital fashion. In spite of this, the development of the fashion industry is always characterized by a mixed bag of risks and opportunities, which is consistent with a neutral attitude.

## Discussion

This study attaches importance to the collection of user information and systematical analysis on the development status, impact and future trend of digital fashion in the industry employing UGD. There have been similar studies conducted in the field of fashion with the following research methods, such as Vãtãmãnescu et al. (2021), which used a questionnaire-based survey to collect data and combined structural equation modeling (SEM) to analyze the data (2021). The systematic literature review (SLR) method and statistical analysis method were used to analyze some text-based information (Jain et al., 2022). The difference with other fashion field related studies is that the research methods used in this study, as well as the perspectives adopted in the analysis on the problem, is the verification of multiplicity.

This study confirms the validity of computer algorithms and computer informatics for analyzing audience perception. Using LDA topic modeling, K-means clustering analysis, and semantic network analysis, the research collected and summarized data in this study. There is innovation in the collection of user information, which leads to fast, massive, and easy access to relevant information (Sheehan, 2002) so as to reason and analyze user cognition and behavior in the digital ecosystem. It has been shown that by combining computer information technology with qualitative analysis methods, text-based information can be processed effectively as well as explored in depth (Ren et al., 2021; Hopkins, 2022; Kliestik et al., 2022; Nica et al., 2022b).

In accordance with the findings, there are a large number of posts and a wide range of topics related to digital fashion and virtual fashion. For now, the discussion’s content is primarily reflected in news articles regarding the creation and sale of relevant brands and related content, such as virtual clothing, virtual idols, virtual beauty, virtual accessories, and other works of art, as well as the impact of digital fashion on the industry, the modification or subdivision of enterprise marketing strategies, and attitudes and evaluations of this phenomenon. The studies and perspectives from Prakash et al. (2020), Wu et al. (2022) and Xue et al. (2020) can prove the validity of the results of this study.

The specific manifestations and new findings are:

### Text analysis—Information mining—Keyword extraction—Industry trend prediction

Based on the data analysis, high-frequency words such as “computer,” “photography,” “metaverse,” “coin telegraph,” and “NFTs” are extensively discussed. It is the former that represents technological progress and innovation underlying digital fashion, while the latter is a reflection of the interaction between fashion trends or market development trends, economic models, and marketing strategies. According to Kamel and Mohamed (2021), the fashion industry should pay attention to innovation in marketing and technology strategies as well as innovation in product design in the context of digitalization. Demand-driven models’ radical business models and digital innovation may be critical to the transformation of the fashion circular economy.

### Text analysis—Information mining—Sentiment judgment—Market judgment

The results of a semantic network analysis indicate that the majority of the audience has a positive and neutral attitude toward digital fashion (Park and Kang, 2021; Breiter and Siegfried, 2022; Toraman, 2022). By using computer algorithms and computer informatics to analyze the lexical and textual content of UGD, it is possible to derive the audience’s attitude toward a topic or event. As a result of the research conducted by Aburbeian et al. (2022) perceived curiosity and perceived pleasure are positively associated with perceived ease of use (2022). As an emerging topic, it has aroused the curiosity and attention of the public. A minority of individuals express negative emotions and rejection. Shen et al. (2021) also expressed similar views in their research, arguing that consumers’ purchasing desires and purchasing behavior need to be improved in an immersive virtual reality environment (Ning et al., 2021; Shen et al., 2021).

### Text analysis—Information mining—Information integration—Strategy adjustment

The posts about digital fashion contain a lot of information and cover a wide range of topics. According to Sayem (2022), this result was confirmed in his study. According to reports, the digital fashion trend has been covered across the board, including: (1) digital design and e-prototyping; (2) digital business and promotion; (3) digital human and metaverse; and (4) digital apparel and smart e-technology (2022). The majority of the keywords are nouns and lexical terms, and the theme of each cluster is independent and pluralistic, indicating that users’ information demands under the topic have obvious similarity and are difficult to completely satisfy (Xue et al., 2020; Damar, 2021; Aburbeian et al., 2022), and that such topics continue to be hotly debated, largely because consumers and professionals are interested and curious.

A critical perspective is required to assess the impact of digital fashion on business. Digital technology is permeating both the upstream and downstream of the fashion industry’s supply chain, maintaining the link between user demand, goods and services supply, and the links (Ning et al., 2021; Moon, 2022). In the stock and rapidly differentiated market segments, the survival and growth of lean and specialized enterprises with “efficiency first” cannot be separated from digitization, and digitization will be beneficial to the vast majority of businesses (Jeon et al., 2022; Vidal-Tomás, 2022).

However, this effect will accelerate differentiation and eliminate enterprises and labor forces with low viability, resulting in the majority of businesses being at risk of closure (Sayem, 2022). In addition to the acceleration of enterprise differentiation, the bipolarization trend in consumption will become more prominent, and the already fragile fashion industry will be adversely affected by the global pandemic. Due to the deterioration of the economic climate, the incomes of most consumers will be impacted, particularly those in the middle segment, and the bi-polarization of high-end consumption and civilian consumption will become more apparent. Additionally, companies and brands must possess technical skills in software, the Internet, computers, big data, etc., in order to realize digital development. (Campbell and Jovanovic, 2022; Lim et al., 2022), as well as artistic talent and enterprise management talent (Kim, 2021). Consequently, digitalization is the technology enabler of the industry’s general trend, and it will be more urgent and significant than product and consumption upgrades (Melnikova, 2021); therefore, it will be a prerequisite for businesses to take advantage of future market opportunities. It is important to note that the sharp trend of consumption classification not only provides an opportunity for enterprises to develop leanly, but also provides the technical conditions for optimizing and upgrading their operations (Rehm et al., 2015; Bratu and Sabãu, 2022). A digitally enabled environment allows enterprises to increase output and operational efficiency (Joy et al., 2022; Morse and Stein, 2022).

As opposed to previous studies, this study’s results can be summarized into three aspects: microscopic, mesoscopic, and macroscopic, all of which are supported by previous research. The meta-universe can provide further insight into the impact of digital fashion development on aesthetics, consumption habits, and consumption patterns of the general public, while the microscopic can provide further insight into consumption patterns. Along with the consumption of physical fashion items, the transaction of relevant virtual and digital fashion collections expands the traditional method of purchasing and the form of goods (Hassouneh and Brengman, 2011). A mesoscopic impact of the digital fashion concept has been experienced by the fashion industry in terms of its creation and development, which has led to the addition and modification of design methods, design concepts, marketing methods, and marketing strategies of relevant items, in addition to technological advancements and the upgrading of upstream and downstream chains (Lee, 2021; Sayem, 2022). At the macro-macroscopic level, it involves the interaction between digital fashion and financial markets, the global context and the advancement of science and technology, and the influence it has on the future trade of goods, transactions, and the transformation of industries (Melnikova, 2021; Jenkins, 2022).

## Conclusion

A textual approach is used in this study to quantitatively analyze keywords in social media data in relation to virtual fashion and digital fashion. This study uses text mining, semantic network analysis, topic modeling, and sentiment analysis to quantitatively analyze keywords in social media data. The essence of the connotations represented by the data was refined by computer algorithms for text mining and data analysis. This was conducted by combining current literature research with market cases and expert interpretations. To obtain new interpretations and findings from the text data, qualitative and quantitative research were combined. According to academic research in the field of fashion, virtual fashion and digital fashion are widely practiced in the industry. However, academic research is still lagging behind practical applications and industry developments. While the findings of this study are based on data collected from the perspective of audience perceptions, they are not limited to that. This presentation provides an overview and discussion of the current state of the fashion industry and research status from the micro perspective of the audience. By utilizing this paper, scholars may be able to efficiently familiarize themselves with field knowledge and facilitate strategic adjustments by relevant institutions.

### Theoretical contributions

This study has dual theoretical implications. The first is data-driven innovation research was applied in this research, which previously studies lacked the use of serial computer algorithms and combined with UGD methods to obtain information, and combine qualitative analysis to obtain the substantive content behind the information represented. Future studies can use the methodology proposed in this study as a research basis for similar studies.

Secondly, this study is an open access data obtained on the Twitter platform, and this step can be used as a variable for future studies. That is, the subject of this study is used as a variable, research on different topics with using other statistical methods or data analysis methods. Although this study is essentially exploratory in nature, it can be a guiding reference for future quantitative studies of open-ended data analysis.

In addition to this, the theoretical implications for the progress of research in the field of fashion. Different from other disciplines, research in the field of fashion is always affected by market changes, and is more practical disciplinary, and lacks progressive theoretical development.

Dual theoretical implications are available in the results obtained from this study. Firstly, it is about the method of open innovation in research. Previous studies are lack of the deep textual meaning to obtain information and analyze data using serial computer algorithms and computer informatics, as well as the combination with UGD methods. On the theoretical level, future research conduct similar research using the methods proposed in this study as the research basis.

Secondly, this study is about the acquisition of open data obtained from the platform Twitter, which can be used as a variable for future research. In other words, with the theme of this study as the variable, corresponding operation and reasoning are conducted combined with other statistical methods or data analysis methods, or the related research methods of this study. Although this study has the exploratory nature, it can be used as an attempt to conduct quantitative research on open data in the future.

In addition, it has theoretical significance in terms of the research progress in the field of fashion. Different from other disciplines, the research in the field of fashion is often subjected to the influence of the changes in the market and has strong practicality, and it lacks step-by-step theoretical development. Through data mining in a large amount, this study systematically introduced and analyzed the development and trend prediction of “digital fashion,” in combination with market reports and industry information, and put forward the multi-faceted impact of “digital fashion” on individuals, society, industry and industry on the basis of objective facts, which provides related research with a lot of reference.

### Managerial contributions

From a more practical point of view, the research is very useful for the industrial development. Designers, individuals, companies, organizations and industries can make adjustments to strategies by referring to our research, which will promote the development of open innovation in the fashion industry.

In addition, This study provides the following examples for enterprises and brands development: (1) Marketing and product promotion strategies must emphasize communication with customers, which is essentially reflected in communication channels and emotional communication. Focus on online marketing, creating topics and events that will appeal to the general public and target consumer groups. Emphasizing the emotional needs of the consumer, building an emotional connection with the public in order to obtain the brand premium. (2) The analysis of text using computer algorithms can be useful for evaluating market dynamics for companies or brands. In quantitative text analysis, data resources can be collected comprehensively for a certain topic, and effective information can be summarized. The company or brand can then respond quickly to the market, adjust the brand strategy, design strategy, or update the brand connotation and value appeal as necessary. (3) Digitization represents the technology enabler of the industry’s general trend, and it will be more urgent and significant than product and consumption upgrades. Therefore, it will be a prerequisite for businesses to participate in the future. The sharp trend of consumption classification not only provides an outlook for the lean development of enterprises, but it also provides the technical conditions for their operation optimization and upgrading, enabling enterprises to increase their output and operational effectiveness under digitally enabled conditions.

### Limitations and future research

Despite the fact that the study provided some useful information to the existing literature, it has some limitations that can be addressed by future research. In this study, text documents of posts from social networks and online communities were gathered. However, the first limitation is related to methodology. In this process, the search and collection of content are conducted based on the keywords on the platform Twitter, as well as the collection of text information, but research on other platforms is not conducted. It is mainly for the reason that the research methodology is subjected to the limitation of requirements on the open platform, and in further research, it is necessary to validate our findings on data from other major social networking platforms.

Secondly, there was no in-depth exploration of the reasons audiences have negative, positive, and neutral attitudes toward digital fashion respectively, nor what their specific concerns, real needs, or psychological demands are. A more in-depth exploration of this issue is necessary and may be the subject of future research.

## Data availability statement

The original contributions presented in this study are included in the article/supplementary material, further inquiries can be directed to the corresponding author.

## Author contributions

YZ conceived the ideas, experimental design, data analysis, interpretation of the results, and drafted the manuscript of the analysis. D-BL gave technical guidance, provided continuous support to perform the experiment successfully, and gave the suggestions about the writing. SL offers suggestions about the manuscript. All authors read and approved for the publication.
